# Integrative taxonomy in helminth analysis: protocols and limitations in the twenty-first century

**DOI:** 10.1186/s13071-025-06682-6

**Published:** 2025-03-05

**Authors:** Alicia Rojas, Laura G. Bass, Josué Campos-Camacho, Fernando A. Dittel-Meza, Cristian Fonseca, Ying Yi Huang-Qiu, Roberto W. I. Olivares, Luis M. Romero-Vega, Fabián Villegas-Rojas, Alberto Solano-Barquero

**Affiliations:** 1 Laboratory of Helminthology, Faculty of Microbiology, University of Costa Rica, San José, Costa Rica; 2 Centro de Investigación en Enfermedades Tropicales, University of Costa Rica, San José, Costa Rica; 3 Laboratorio de Patología Veterinaria LAPAVET-ESFA, Catedra de Patología e Histología, Escuela de Medicina y Cirugía Veterinaria San Francisco de Asís, San José, Costa Rica; 4 Laboratorio Institucional de Microscopía, Instituto Tecnológico de Costa Rica, Cartago, Costa Rica; 5 Pathology Department, School of Veterinary Medicine, Universidad Nacional, Heredia, Costa Rica

**Keywords:** Helminthology, Molecular analysis, Morphometry, Histopathology, Ecology, Epidemiology

## Abstract

The term integrative taxonomy was coined in 2005 for the identification of microorganisms using morphological, molecular, pathological and ecological components. Since then, more than 200 scientific articles have been published using this term in the helminthology field in different geographical regions of the world, leading to accurate identifications of specimens and expanding the known biodiversity by describing novel species. Importantly, the responsible use of this approach has led to balanced results in which molecular data do not overshadow morphological, pathological or ecological information. By having different perspectives, new taxonomic situations have arrived including the definition of cryptic diversity, species complex and genotypes. In the present work, we present the main procedures and principles for conducting an integrative taxonomy study of parasitic helminth including collection and preservation of specimens, fixation and staining of worms for light microscopy and scanning electron microscopy, and histopathological, ecological and DNA analysis. This guide is aimed at researchers in their endeavor to analyze helminth parasites collected from wild or domestic animals with examples provided, but the protocols presented herein may be extrapolated for the analysis of helminths collected from other hosts (e.g. human or fish). One important aspect is that the recommendations presented herein derive from the authors' experiences, which will hopefully be of use to the reader.

**Graphical Abstract:**

**Figure:**
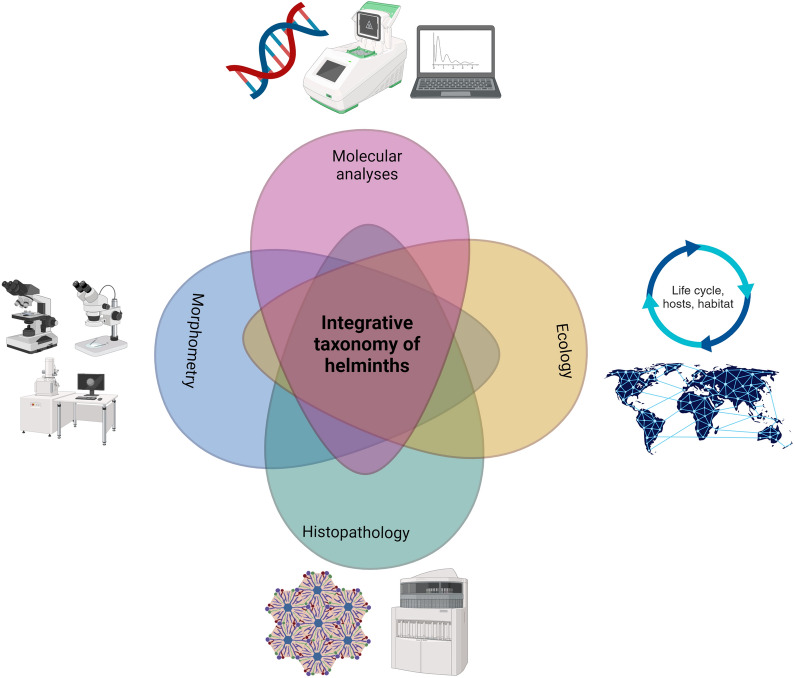

**Supplementary Information:**

The online version contains supplementary material available at 10.1186/s13071-025-06682-6.

## Background

**Fig. 1:**
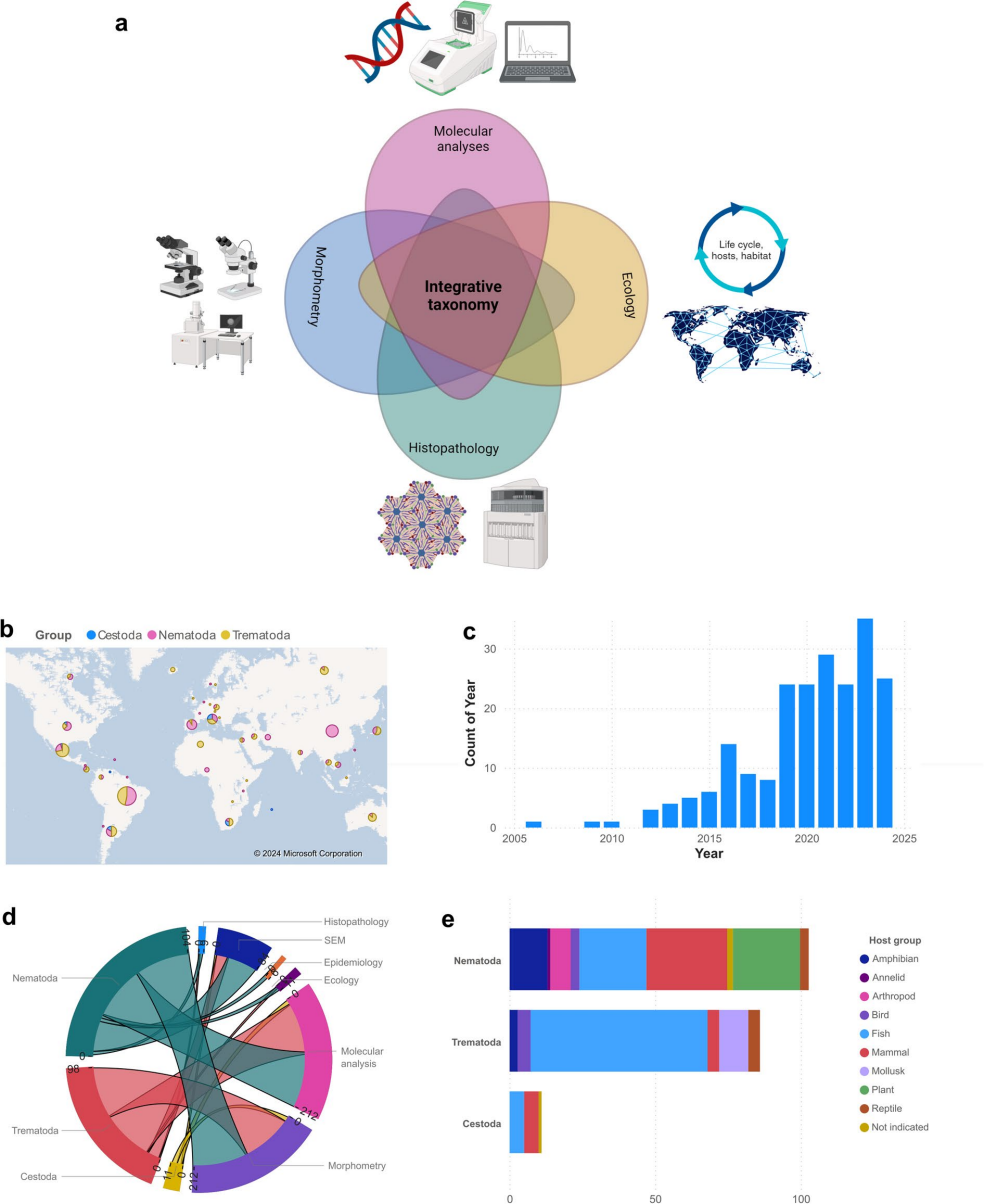
Integrative taxonomy philosophy and its use since 2005. **a** Diagram of integrative taxonomy approaches and use of this approach in the study of helminths since the introduction of the term in 2005. This figure was created using Biorender.com. **b** Number of publications per country using “integrative taxonomy” terms in their title, abstract or keywords in NCBI. Bubble size is proportional to the number of publications, and pie charts relate to the helminth group. **c** Number of publications per year using “integrative taxonomy” terms in their title, abstract or keywords in NCBI. **d** Integrative taxonomy approaches used in nematodes, trematodes and cestodes. **e** Host groups in the study of nematodes, trematodes and cestodes

Taxonomy is central for species identification and delimitation and therefore for evaluating and understanding the biodiversity of our planet [1]. This science has traditionally been carried out using comparative morphology, but with the advent of molecular tools and the low price of obtaining DNA barcodes, some debate has arisen on whether molecular analyses will replace traditional systematics [2]. This is why the term integrative taxonomy was introduced in 2005 [2, 3] and has been increasingly used in the last 20 years in the fields of zoology [1], botany [4], entomology [5], mycology [6] and parasitology [7, 8]. When applied to parasitology, integrative taxonomy refers to the complementarity of different disciplines in the classification of organisms [1], for instance, (i) the evaluation of morphological characteristics of specimens by using light and scanning electron microscopy, (ii) histopathological analysis of parasites and the lesions induced by them in their hosts, (iii) ecology and epidemiology using different habitats and hosts and research on the geographic distribution of pathogens and (iv) DNA barcodes and their phylogenetic study (Fig. 1a). There is ongoing debate regarding how to apply evidence to delimit species [1]. Nevertheless, integrative taxonomy has improved our understanding of species limits and the way new species are described.

Integrative taxonomy has been applied to different fields of parasitology, where sand flies as vectors of *Leishmania* spp. have been correctly characterized for epidemiological purposes [9], the *Echinostoma “revolutum”* complex detangled [7] and the presence of potential new species or cryptic diversity in helminths found [10, 11]. In this sense, the field of helminthology has suffered several transformations: from the sole analysis of morphometric characters and the organism's ecology to the incorporation of DNA barcodes [12]. The divorce between morphological, ecological and molecular methods [5] has led to inaccurate descriptions, a vast number of DNA sequences deposited in databases with incomplete or wrong identifications and inflation of the number of new species [13]. Therefore, integrative taxonomy enriches the complementarity of each discipline in the analysis of helminth specimens [14]. Clearly, this approach requires a multidisciplinary team composed of biologists, veterinarians, physicians, microbiologists and other professionals, including engineers who may not be directly related to biology but are experienced in the technical operation of complex equipment.

The field of helminthology studies organisms of the phyla Nematoda and Platyhelminthes, which include the classes Trematoda and Cestoda [15]. Organisms of other groups like the class Acanthocephala or Pentastomida have suffered several taxonomic reclassifications and therefore have intermittently been members of the vast group of helminths [13]. As of mid-2024, articles available in NCBI with “integrative taxonomy” terms in their title, abstract or keywords included 104 papers dealing with nematodes, 98 with trematodes and 11 with cestodes (Supplementary file 1). In this analysis, it is shown that Brazil (21 articles with trematodes and 24 with nematodes), Mexico (5 with nematodes, 15 with trematodes and 1 with cestodes) and China (18 with nematode studies) have the most articles with this terminology (Fig. 1b). In addition, 47.89% of the studies were conducted in the Americas, followed by Asia (20.19%) and Europe (17.84%), and the number of papers with this approach steadily increased from 2005 until mid-2024 with 25 published papers (Fig. 1c). Moreover, most articles use morphometry, molecular methods and scanning electron microscopy (SEM) for their analysis, whereas histopathological characterizations, epidemiological or ecological factors are less considered for specimen identification (Fig. 1d). Interestingly, the hosts from which the articles are concentrated vary between helminth groups. For nematodes, mammals, plants and fish are the most studied hosts. In contrast, > 50% of studies on trematodes focus on fish hosts, while cestodes have been analyzed in both fish and mammal hosts (Fig. 1e). These data demonstrate that integrative taxonomy in helminthology has been increasingly used through the years in different regions of the world and in different host species. However, researchers need guidance on general procedures and technical considerations.

Herein, we present the principles and main procedures for the analysis of helminth parasites when using an integrative taxonomy approach. This work does not aim to be a comprehensive review of all available methods for studying helminths but provides guidelines and protocols useful for those researchers carrying out analyses of specimens isolated from mammal hosts. Nevertheless, some of the procedures mentioned here may also be applicable to studies in helminths associated with other host groups, such as fish, insects or even plants. For instance, helminths collected from fish are likely to follow the same pipeline suggested in this work if host and helminth samples are collected, preserved and processed appropriately. In addition, worms associated with plants or insects may also be studied with the recommendations suggested in these guidelines, but modifications to the histopathology or SEM analysis should be incorporated. Finally, some procedures explained later, such as sieving gastrointestinal contents to collect large amounts of specimens, may not be applicable to human hosts, as people today are not as highly parasitized as wild or domestic animals. Therefore, not all procedures will be suitable for all host sources, and some modifications will be found elsewhere to fill these gaps.

### Specimen collection and preservation

Specimen collection will depend on the animal species (i.e. wildlife or domestic species), organ affected and animal condition (*ante mortem* vs *post mortem*). For instance, domestic species can be more easily examined for parasite presence through physical examination, coprological analysis and diagnostic imaging. These procedures can also be applied to zoo or confined species. In contrast, sampling free-ranging hosts normally involves capture and release procedures, where the parasitological examination cannot be as detailed [16]. According to our experience, free-ranging animals are best examined by performing necropsies. Nonetheless, this is not always possible due to ethical and species conservation concerns. On the other hand, helminth specimens can be collected in live animals by surgery depending on the affected tissue. For instance, *Dirofilaria immitis* can be extracted by transvenous extraction [17], encysted larvae or metacestodes using nodule surgical removal (e.g. hydatid cysts or cysticerci) [18] and gastrointestinal larvae or adults like *Cylicospirura* spp. through enterotomy [19]. Other less invasive procedures, such as endoscopy, are optimal for the upper gastrointestinal tract, such as for the collection of *Spirocerca lupi* worms in esophageal nodules [20]. Also, antiparasitic drugs are useful for the collection of live gastrointestinal helminths like *Dipylidium caninum*, hookworms or *Toxocara* spp.

**Fig. 2:**
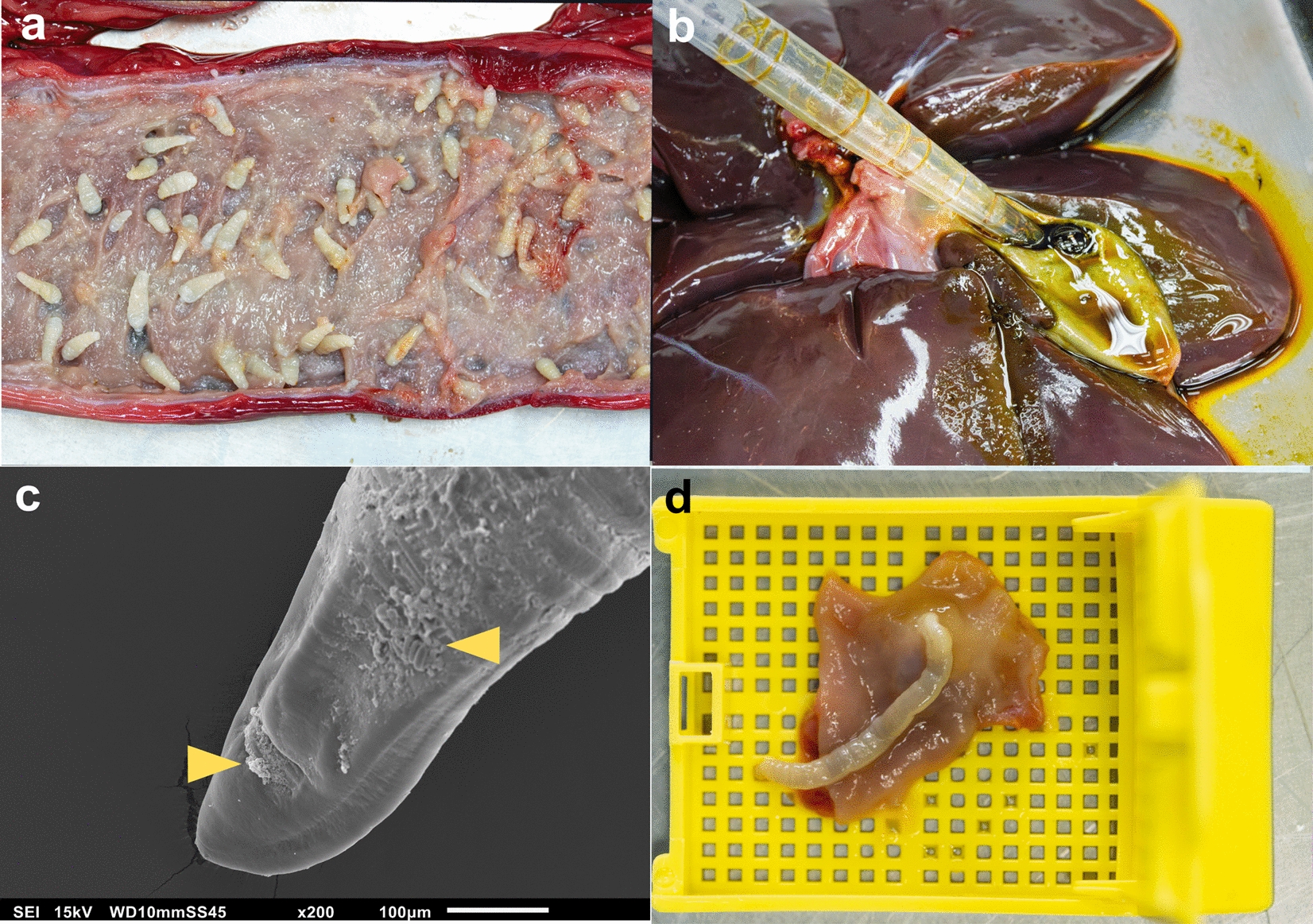
Collection of specimens from different samples and uses. **a** Direct visualization of *Oncicola venezuelensis* on a jaguar (*Panthera onca*) colon. **b** Collection of *Controrchis caballeroi* using plastic Pasteur pipettes on a mantled howler monkey (*Alouatta palliata*). **c** Observation of host tissue remnants in the surface of a *Cylicospirura* sp. (yellow triangle) collected from a yaguaroundi (*Puma yagouaroundi*). **d** Placement of a *Prosthenorchis elegans* from a squirrel monkey (*Saimiri oerstedii*) in a plastic cassette prior to fixation

On routine necropsies, helminths are normally collected by direct visualization, which can lead to overlooking smaller specimens. Therefore, careful examination and collection should be aimed whenever possible to assess the true helminth diversity. Solid organs can be washed over a 106-µm siege using running tap water [21]. This method is rarely used on standard necropsies and is most used on helminth-focused post-mortem studies. In addition, soaking tubular organs in saline solution at 37 °C can aid in the retrieval of specimens, such as in the abomasum of small ruminants [22] (Fig. 2a). Then, tubular and cavitary organs, like the gallbladder, must be carefully opened using scissors [21] (Fig. 2a). The bile content can be extracted using plastic Pasteur pipettes and placed in a petri dish to collect the parasites directly, for instance *Fasciola hepatica* (Fig. 2b). Large parasites can be collected from the organs under direct visualization.

Live specimens should be relaxed prior to their fixation when morphometric analysis is carried out using light microscopy or scanning electron microscopy (SEM). This is accomplished by placing live specimens in warm (37–42 °C) saline solution or PBS for 8–16 h until they lose their viability. Then, it is recommended to clean the parasites from the host's tissues using a soft brush. Otherwise, tissue remnants will significantly affect the observation of the worm's surface topology and structures in the SEM for further taxonomic placement (Fig. 2c). Subsequently, the parasites should be placed and stretched in a proper position (Fig. 2d); for instance, it is recommended to stretch nematode worms or place trematodes or flukes in a dorsoventral position for their morphological analyses. If histopathological analysis can be performed, the specimens can be placed in warm formalin after their relaxation in saline solution.

The gastrointestinal tract of small animals (birds, bats or rodents) can be extended and pinned prior to fixation to avoid contraction of specimens. This small gastrointestinal tract can also be opened and washed; unfortunately, this can be challenging in field conditions where mist netting is usually performed in birds or bats. Whole fixation in formalin of the organ can also be done, which gives excellent results for evaluating parasite-tissue interactions through histopathology (Fig. 2d), immunohistochemistry, in situ hybridization and, most recently, spatial transcriptomics [23–25]. Nonetheless, formalin fixation is suboptimal for molecular identification since it reduces DNA quality. Molecular identification using formalin-fixed paraffin-embedded (FFPE) tissues has been useful in retrospective studies [26].

Placing helminth specimens in distilled water or other hypotonic solution induces the massive release of eggs from the uterus [27]. This eases subsequent staining, observation of inner structures and collection of eggs for their morphometric analysis. For instance, placing the human lung fluke *Paragonimus mexicanus* in water for 1–2 h leads to the release of eggs to the solution, thus facilitating next steps. Eggs may be collected from the fluid in which the worms were collected. Live specimens in distilled water their uterus with the subsequent release of large amounts of eggs. These eggs suspended in water or the carrying fluid can be concentrated by centrifuging the tube for 10 min at 2000×*g*. Then, the upper fluid is discarded, and the eggs may be resuspended in formalin or PBS, depending on the purpose. If molecular analyses are done, eggs should be resuspended in a small volume of PBS, but if eggs are to be kept as part of a collection, then long-term storage in formalin at 4% or 70% ethanol is recommended [27]. Analysis of larvae may follow the same procedure, and they should be kept in formalin at 2% or 70% ethanol for long-term storage.

### Fixation and staining of specimens

The specific protocols, including reagents, incubation times, mix proportions and omission and addition of steps, vary from laboratory to laboratory, as each analyzer provides their own expertise to the method. In addition, differences between helminth group characteristics also lead to protocols tailored to each need. Traditionally, nematodes are cleared in lactophenol or lactophenol of Amann [28], phenol-alcohol, glycerin or Beechwood creosote and mounted in glycerin, Canada balsam or Permount to allow morphometric observations [29]. The thick and impermeable cuticle of nematodes does not allow proper staining, and special protocols are used for this purpose, such as Semichon's carmine [29]. Nematodes are usually cleared in any of the above solutions for 24 h, but in our experience, thinner roundworms like filarioids should be incubated for a few hours since longer periods may lead to complete clearing of inner and outer structures and impairment of proper taxonomical classification. The original recipe of lactophenol consists in the mix of one part lactic acid, one part phenol crystals, two parts glycerol and one part distilled water [30]. Glycerol clears and prevents the drying of specimens, while lactic acid and phenol clear the cuticle of nematodes while keeping inner structures still visible [27]. Temporary mounts of nematodes can be done in lactophenol, and permanent mounts are usually kept in glycerin in properly sealed slides [31].

**Fig. 3:**
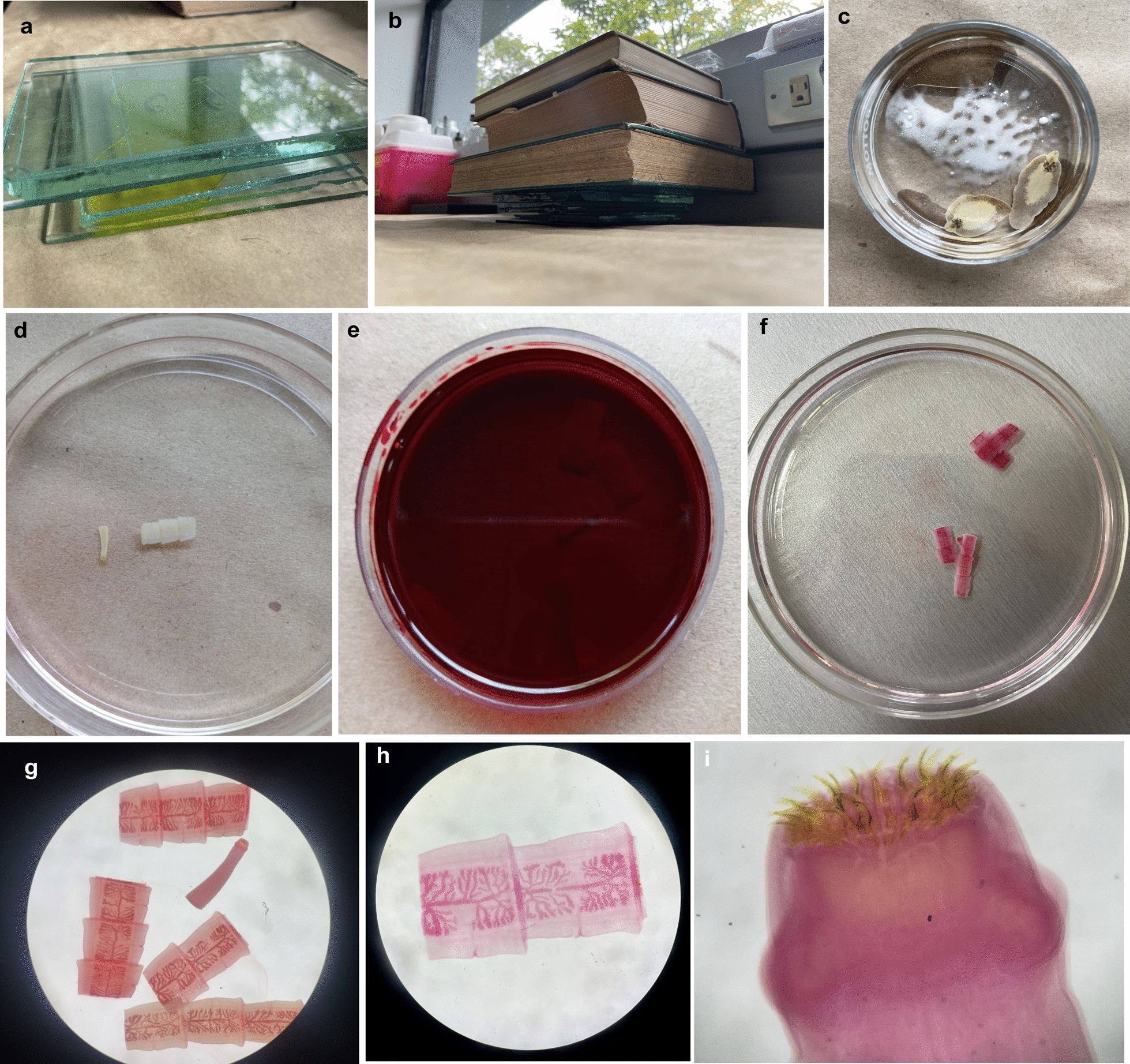
Main steps during the hydrochloric carmine staining protocol for trematodes. **a** and **b** Fixation and flattening of a *Fasciola hepatica* specimen using Bouin's solution, glasses and books. **c** Addition of lithium carbonate to the specimens to increase the solution's pH in a 6-cm petri dish. **d** *Taenia pisiformis* scolex and proglottids stained in ethanol washes to remove excess lithium carbonate in a 6-cm petri dish. **e** *Taenia pisiformis* scolex and proglottids stained with hydrochloric carmine. **f** and** g** Diafanization of worms with methyl salicylate. Picture taken in a 6-cm petri dish. **h** *Taenia pisiformis* mounted in Canada balsam (scale bar = 0.5 cm). **i** *Taenia* sp. scolex mounted in Canada balsam (scale bar = 70 µm)

Carmine is the most employed compound when staining is possible in the laboratory. Several variations have been described based on the same dye, such as carmine-propionic acid, Semichon's carmine or acetocarmine, hydrochloric carmine, Schneider's carmine and Belling's carmine [32] (Fig. 3). As above, each laboratory may find a particular protocol with its respective modifications useful. For instance, Semichon’s and carmine-propionic acid stains have been used for nematodes [33] or larval or small adults of trematodes and cestodes. In our experience, the hydrochloric carmine staining procedure is the easiest to follow since the reagents are readily available, and the best results are obtained in adult trematodes and cestodes. Carmine derives from cochineal hemiptera dyes and was used in ancient civilizations of the Americas [34]. Carmine binds to glycogen, mucin or DNA through its aluminum ion within the carmine molecule by coordinate or hydrogen bonding depending on the stain's formulation, pH and ion content [34].

The protocol provided in Box 1, known as hydrochloric carmine, is used to stain trematodes and cestodes (Fig. 3). When carmine is used with lithium carbonate, a high pH solution is formed; therefore, nuclei and cytoplasm are successfully stained. Then, acidic alcohol clears the cytoplasm by the dissociation of carmine from the carboxyl groups in the cytoplasm and only phosphoric acids of nucleic acids retain the stain [34]. At least ten specimens of each sex should be stained or cleared for their morphometric study so their mean and standard deviation values can be estimated for each structure. In addition, the study of several worms of the same morphotype allows the visualization of most characteristics that may not have stained or been correctly observed in some specimens. Importantly, adult stages are usually required to successfully identify a specimen as these have fully developed sexual organs, which are typically the key characteristics listed in taxonomic keys, as opposed to metacestodes, asexual stages of trematodes or nematode larvae. Furthermore, according to our experience in identifying nematodes, male adults are usually more informative than females since the former have more structures useful for their classification.

Box 1. Hydrochloric carmine staining protocol for trematodes and cestodes **Reagents**

- Bouin's solution: 75 ml saturated picric acid solution, 25 ml 40% formalin, 5 ml glacial acetic acid
- Hydrochloric carmine: 5 g powdered carmine, 5 ml concentrated HCl, 5 ml distilled water, 200 ml 90% ethanol. Mix, boil in a water bath for 1 h and filter
- Lithium carbonate
- 70% ethanol
- 0.5% acidic alcohol: 100 ml 70% ethanol and 0.5 ml concentrated HCl
- 95% ethanol
- Absolute ethanol
- Pure methyl salicylate

**Procedure**

i. Fix the specimens with Bouin's solution for 24 h: Place the specimens on top of a thick glass and immerse them in Bouin's solution. Place another glass on top and gradually add weight until the specimens are flattened (Fig. 3a and b).
  ● Be sure the specimens are completely immersed in Bouin's solution and no bubbles have formed around them.
ii. After fixing the specimens, remove the glass covers and carefully detach the specimens to prevent their breakage.
iii. Transfer the specimens to a petri dish with Bouin's solution only (for at least 4 h).
iv. Then, move the worms to a petri dish with distilled water and agitate them to wash off excess Bouin's solution.
v. Transfer the specimens to a petri dish with 70% ethanol to remove the excess Bouin's solution.
vi. Move the worms to a petri dish with 70% alcohol and add a small amount of pure lithium carbonate to remove Bouin's solution. Lithium carbonate will eventually mix with the 70% ethanol.
vii. Once the specimens are white, remove the specimens from the lithium carbonate. This can take a couple of days, but agitation can speed up the process.
viii. Transfer the specimens to 70% alcohol to remove lithium carbonate. This step can be repeated several times using a fine brush or wooden stick (Fig. 3d).
ix. Place the specimens in hydrochloric carmine in a petri dish or Eppendorf tube for 10 to 30 min, depending on specimen size (Fig. 3e).
x. Transfer the specimens to 70% alcohol and perform several washes.
xi. Transfer the specimens to 0.5% acidic alcohol until proper discoloration.
  ● At this stage, careful monitoring is crucial to achieve proper discoloration and contrast. If over-decolorized, specimens may need to be re-stained with hydrochloric carmine.
xii. Transfer the specimens to 70% alcohol and perform two washes to remove excess of acidic alcohol.
xiii. Transfer the specimens to a petri dish with 70% alcohol and add a small amount of lithium carbonate for 10 to 20 min to remove acidic alcohol (Fig. 3c).
xiv. Perform three or more washes in 70% alcohol to remove lithium carbonate.
  ● Lithium carbonate particles should be completely removed by this point.
xv. Place the specimens in 95% alcohol for 4 h.
xvi. Place the specimens in absolute alcohol for 4 h.
xvii. Clarification or diaphanization: Ensure specimens do not float during these steps to prevent them from re-hydrating and turning black (Fig. 3f).
xviii. Mix three parts of absolute alcohol with one part of pure methyl salicylate and place the specimens in a petri dish for 20 min.
xix. Mix two parts of absolute alcohol with two parts of pure methyl salicylate and place the specimens in a petri dish for 20 min.
xx. Mix one part of absolute alcohol with 3 parts of pure methyl salicylate and place the specimens in a petri dish for 20 min (Fig. 3g).
xxi. Mix with pure methyl salicylate for 20 min.
  ● Specimens can remain in this solution until mounting.
xxii. Mount specimens in Permount or Canada balsam, spreading them to the size of the coverslip and avoiding bubble formation.
xxiii. Add a coverslip, seal the coverslip with nail polish and label the slide with the scientific name of the specimen; name of the collector and date and place of collection (Fig. 3h and i).
xxiv. Let the slides dry for several days until the mounting medium is completely dry.

### Scanning electron microscopy (SEM) observations

**Fig. 4:**
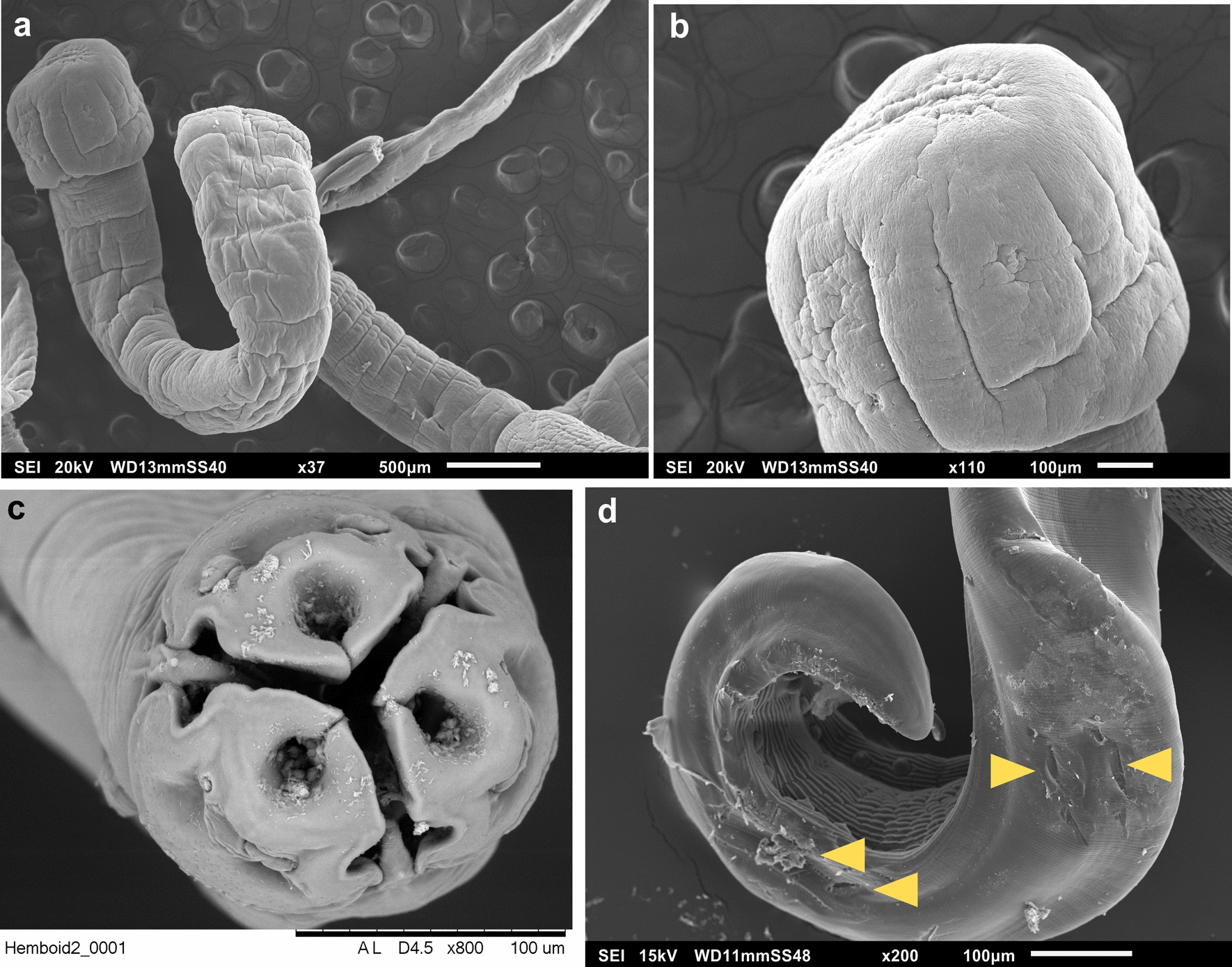
Specimens observed under scanning electron microscopy. **a** *Litobothrium aenigmaticum* collected from the thresher shark, *Alopias pelagicus*. Shown is a section of the scolex and strobila of the tapeworm and in **b** the magnified scolex.** c** Frontal view of a *Lagochilascaris minor* adult collected from a domestic cat, *Felis catus*. **d** The use of tweezers to handle specimens can break the worm's cuticle as observed in this picture

Due to the increased imaging depth of focus and resolution of SEM, several reviews of different methods applied to flatworms and nematodes have been reported since the 1970s. Electron microscopy has been a great tool to analyze regional differences in the worm's surface microtopography, features that could not be seen with a light microscope [35]. Therefore, such observations have increased the resolution to observe structures and has been an essential tool to describe new species and the delimitation of species complexes. Usually, SEM studies are carried out for external topography studies of helminths (Fig. 4a, b and c). However, with more specific methods, such as those described by Adnet et al. [36], the worm's inner organization can also be observed, while preserving their cellular structures for morphology, ultrastructure and taxonomic characterization.

**Fig. 5:**
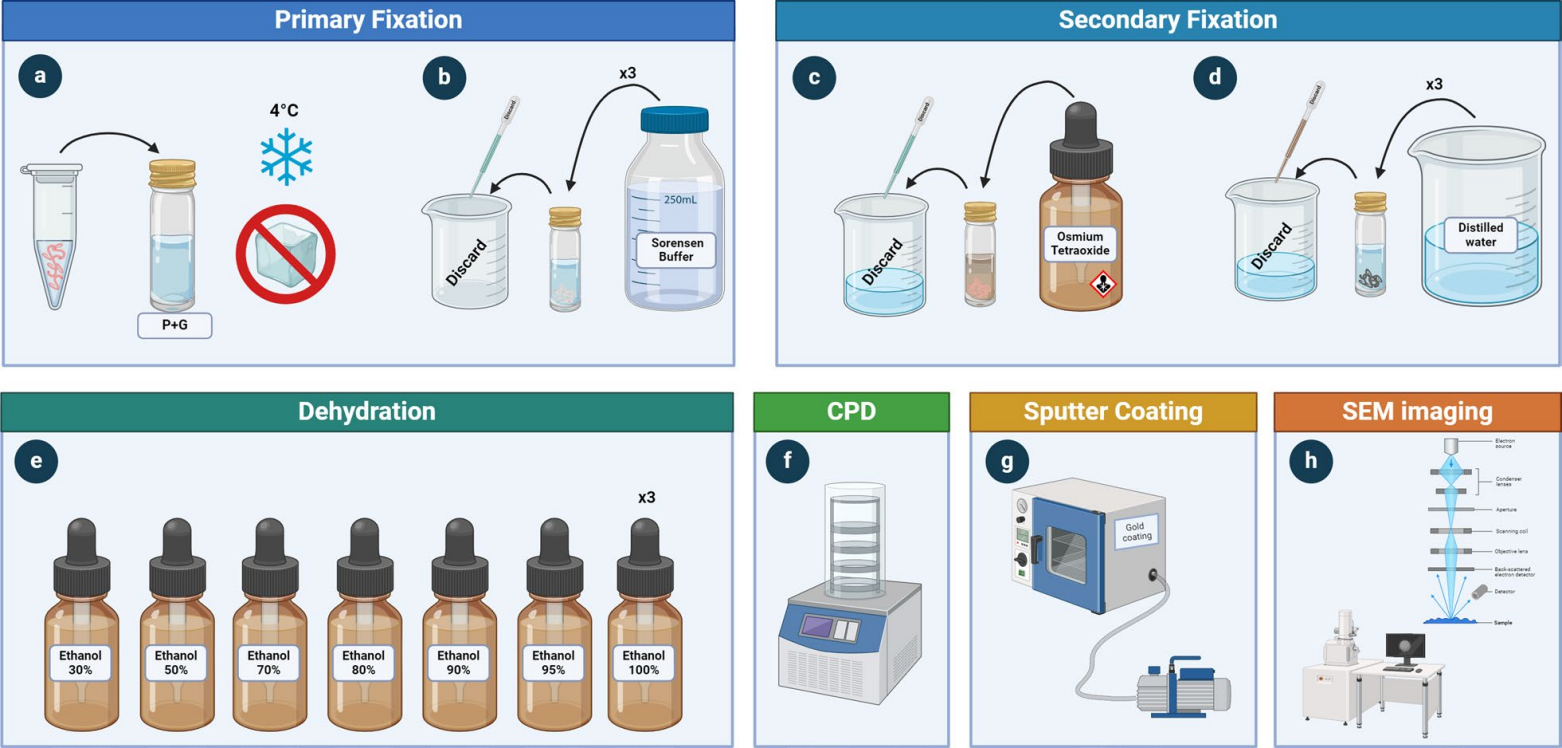
General biological tissue procedure for scanning electron microscopy preparation applied to helminths. This figure was created using Biorender.com

The general pipeline for preparing specimens has specific solutions and approaches, and some of the methods do not work on all worm species, but there are general recipes [37] (Fig. 5). The first proposed procedures for helminth SEM preparation were based on fixation, dehydration and treatment with an antistatic agent (glycerol-KCl), ending with a gold or palladium coating. Nowadays, fixation agents include paraformalin, paraformaldehyde and acetic acid-formalin-alcohol, and dehydration has been done with ascending concentrations of ethanol from 30 to 95%. Finally, ethanol remnants are evaporated with a glycerol solution and finally coated with a vacuum evaporator [38]. A two-step process with a fixative for proteins and a secondary fixative of osmium tetroxide for unsaturated fats has been proposed with good results. The outcome is a method that halts cellular processes and prevents cracks, shrinkage and volume changes during tissue fixation, allowing the specimens to keep their natural shape. Also, buffers can be used to enhance the specimen's chemical fixation, such as phosphate buffer or sodium cacodylate buffer.

Another factor to consider is the handling of the specimens, since the cuticle and tegument of helminths break easily. Specimens can be transferred from one solution to the next one with dental picks to prevent damage. Contrarily, the use of tweezers is not recommended since the tips' pressure can break the worm's surface (Fig. 4d). Micro-pipetting of very small specimens should be done when transferring them between solutions. Moreover, ethanol for dehydration steps and critical point drying (CPD) in freon or carbon dioxide are proposed to avoid the forces of surface tension, which may collapse or deform the specimen [37, 39].

Nowadays, generic procedures for SEM imaging consist of the following steps:

(i) Placing the tissue or specimens in a vial with a fixation agent, such as glutaraldehyde 2.5% and paraformaldehyde 2% in phosphate buffer at pH 7.4 for 24–48 h at 4 °C.
(ii) Wash the specimens three times with Sorensen’s phosphate buffer at pH 7.2.
(iii) Secondary fixation with osmium tetroxide. The sample can be left at 4 °C or in a cool dark room for 2 h, but in some samples this time may be extended for better results.
(iv) Wash the specimens three times with distilled water.
(v) Dehydrate the sample with an ethanol series of 30%, 50%, 70%, 80%, 90% and 95% for 10 min each and three times with 100% ethanol (as fresh as possible to avoid hygroscopic absorption). Then, it is possible to keep the sample in alcohol permanently if needed [37].
(vi) Specimens are dried in a CPD, where CO_2_ replaces the ethanol at critical point conditions with a recommended temperature of 15 °C and pressure of 50 bar. This process creates no surface tension in the specimen to avoid their collapse [39].
(vii) The sample is then placed into stubs for SEM inspection with a double-sided carbon conductive tape in the desired position for examination. Key sections of the specimens are separated from the rest of the body. These are usually the anterior and posterior ends of nematodes, the scolex of a cestode or, depending on the size, the whole body of a trematode. This step should be carefully and gently done to assure the integrity of the structures.
(viii) The sample is placed in an ionic coater or sputter coater, which transforms a non-conductive sample to conductive at the surface for SEM imaging. The sample can be coated with gold or gold-palladium, with a coating thickness from 5 to 10 nm.

### Histopathology analysis of lesions

Identification of parasites from histological sections is relevant for several reasons. First, it is always necessary to co-localize the parasitic agent with a specific lesion to rule out or confirm its pathogenic role. There are numerous conditions in which parasites are present in tissues without constituting a real threat to the host's health. Therefore, it is essential to understand their biology and to determine whether their presence in animal tissues is pathologically relevant. Examples include ruminitis due to *Paramphistomum* spp. in cattle or gastritis due to *Gasterophilus* spp. in horses, which can only harm the host when the number of parasites is extremely high [40]. Other examples include spirometrosis in cats and dogs, which usually leads to no clinical manifestations [41], or *Hymenolepis nana* or *H. diminuta* infections in humans [42]. In addition, muscle infestations by *Sarcocystis* spp. usually do not produce lesions in their hosts with the exception of rare cases of eosinophilic myositis or myocarditis by *Sarcocystis cruzi* [43] in cattle or infestations by *Gongylonema* spp. in ruminants or by *Klosiella equi* in horses, which do not produce clinical illness [44]. These examples demonstrate that identifying the co-localization of a parasite within a relevant lesion is essential for explaining clinical signs and/or death in an animal and for any diagnostic work.

It is also important to emphasize that usually pathology services receive biopsies from clinicians consisting of fragments of skin, liver, brain or other parenchymal organs with nodular lesions, which reveal a parasitic etiology only after histopathological examination. Examples of these conditions include eosinophilic and granulomatous dermatitis caused by *Draschia* spp. or *Habronema* spp. in horses [43], granulomatous lesions caused by *Halicephalobus gingivalis* in horses or *Heterobilharzia americana* eggs in dogs and other mammals, dermatitis caused by *Stephanofilaria* spp. in cattle or *Pelodera* spp. in dogs, swine and cattle species [40], and pneumonias caused by *Pneumonyssus simicola* in monkeys, just to mention a few examples [40, 45]. Therefore, it is important for the pathologist to identify different types of parasites in histological sections and, based on their morphological characteristics, identify them at the class level and sometimes even family and genus. Finally, not all tissues are suitable for histopathological analysis in live hosts, like cysticerci in human brain. Therefore, some limitations exist in this stage of the analysis. However, it should be noted that compiling information about the affected organ, parasitized animal species and type of lesion and observing parasites in histopathological sections stained with hematoxylin and eosin can often lead to a non-specific diagnosis. Thus, histopathology should often be complemented with additional tools such as PCR and sequencing of amplicons to achieve a more accurate diagnosis.

Specimen collection for parasite histopathology is the same as that routinely used for processing other animal tissues such as tumors or inflammatory lesions of higher animals. Tissues of interest should be fixed in 10% formalin at a ratio of one part tissue to ten parts of formalin at room temperature. Although fixation time varies depending on the size of the sample, it is usual to maintain samples for 24 to 48 h in 10% formalin at room temperature. Using 10% buffered formalin is not essential, but it is recommended in order to avoid artifacts that may interfere with microscopic observations. These fixed samples are then dehydrated with alcohol dilutions of increasing concentration, rinsed with xylol and embedded in paraffin. These FFPE blocks are then cut to obtain 4-µm-thick sections and stained with the routine hematoxylin and eosin staining technique [46].

**Fig. 6:**
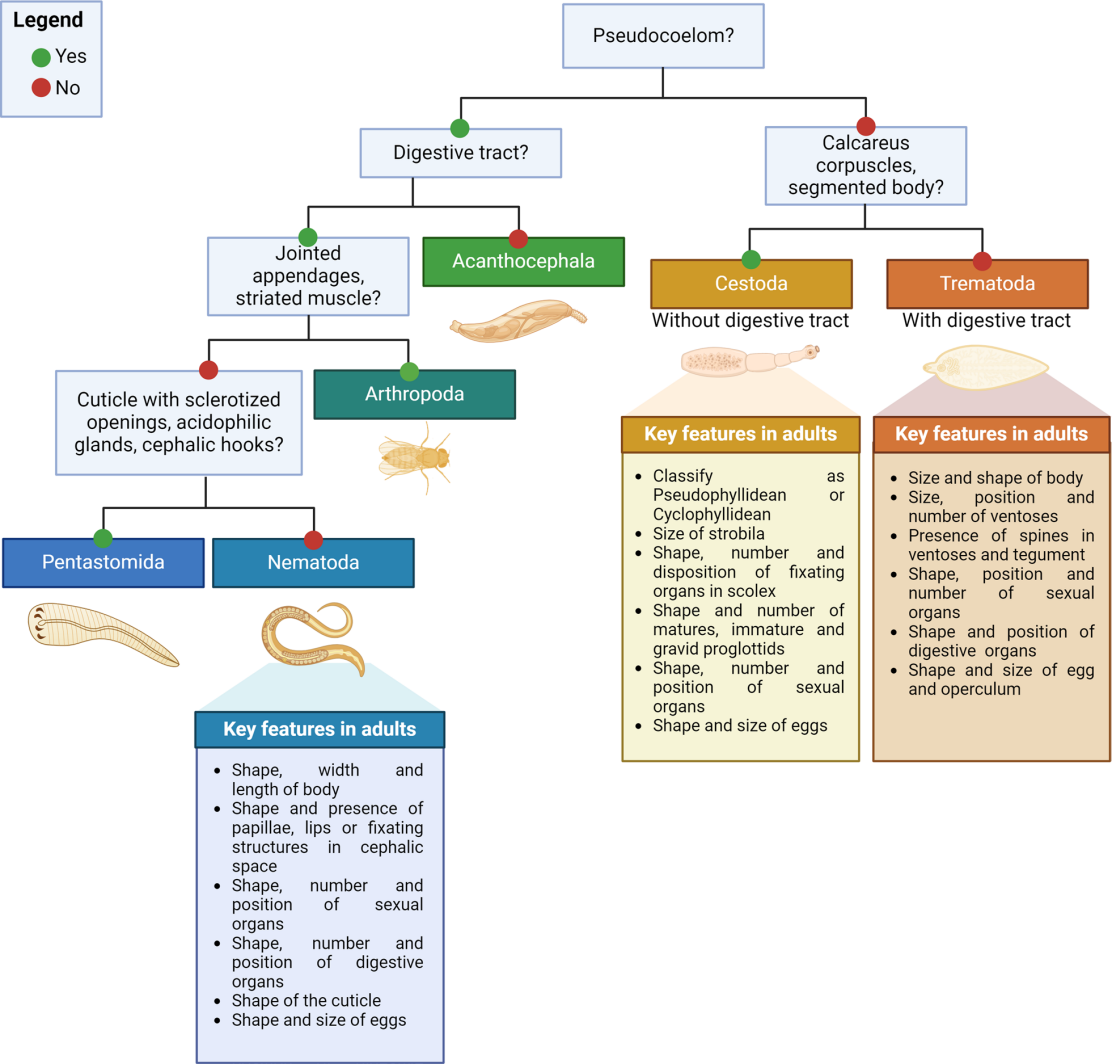
Decision flowchart for identifying parasitic worms during histopathological analysis and main characters of nematodes, trematodes and cestodes to consider during morphological classification. In addition, the main morphological characters of nematodes, trematodes and cestodes for their classification are highlighted. This figure was created using Biorender.com

**Fig. 7:**
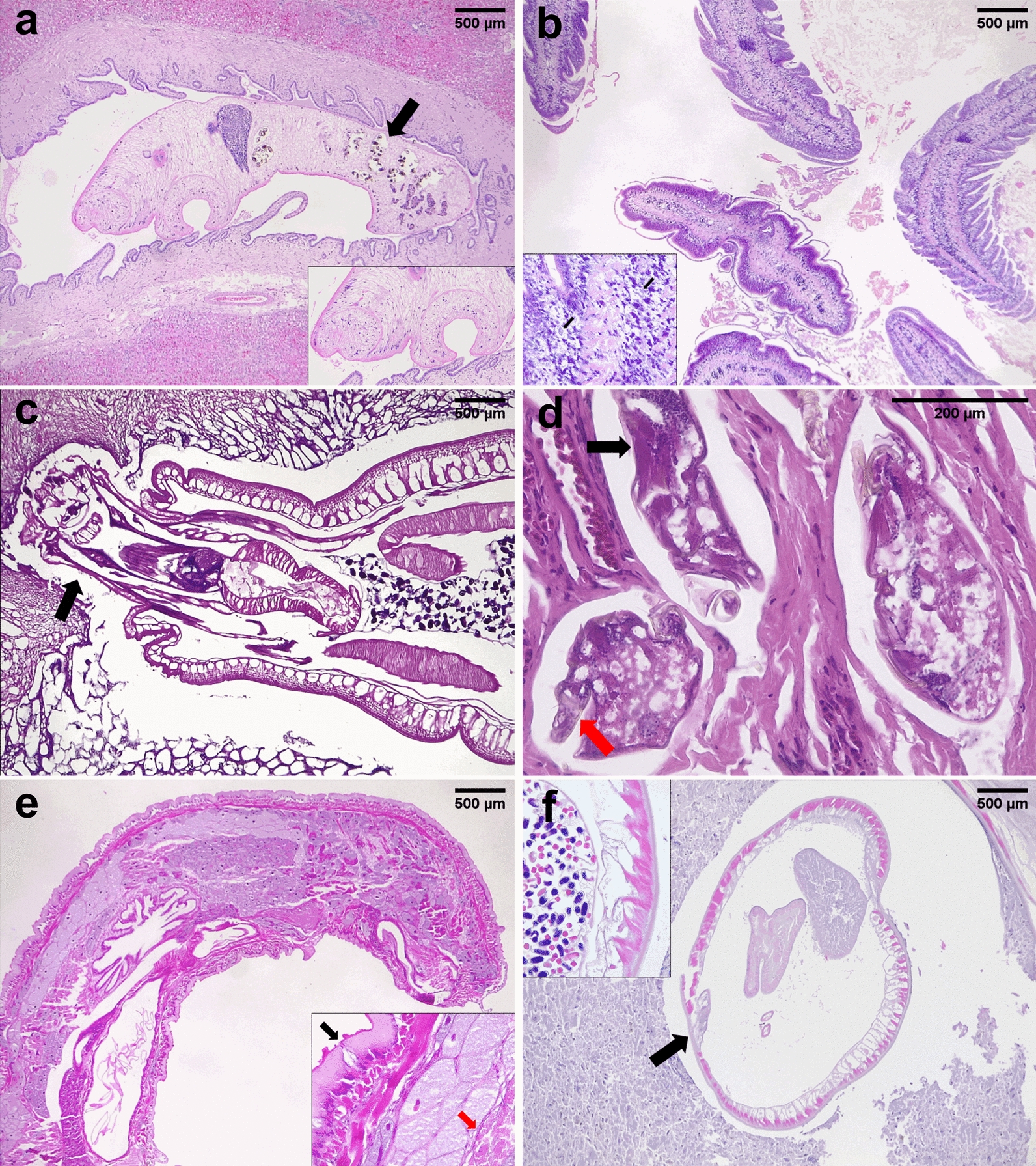
Histopathological analysis of nematode, trematode, cestode, arthropod, pentastomid and acanthocephalan specimens collected from different animal species. **a** *Platynosomum illiciens* is covered by a tegument, lacks a pseudocoelom and presents a uterus filled with ova (black arrow). H&E 40×. Inset: detail of the ventral and oral suckers from the same parasite. **b** *Spirometra mansoni* (cestode), the parasite is covered by a tegument, lacks a pseudocoelom and presents a segmented body. H&E 40×. Inset: detail of the calcareous corpuscles (black arrows) from the same parasite. **c** *Oncicola* sp. (acanthocephala), the parasite has a cuticle, a thick hypodermis, a pseudocoelom and a spined proboscis (black arrow). H&E 40×. **d** Mite of the family *Epidermoptidae* (arthropod), the mite has a chitinized body wall with striated muscles (black arrow) and jointed appendages (red arrow). H&E 100×. **e** *Porocephalus crotali* (pentastome), the parasite presents a pseudosegmented body with an intestine surrounded by acidophilic glands and sclerotized openings in the cuticle. H&E 40×. Inset: detail of the acidophilic glands (red arrow) and the sclerotized openings in the cuticle (black arrow) from the same parasite. **f** *Spirocerca lupi* (nematode), the parasite presents a cuticle, a pseudocoelom, coelomyarian musculature and lateral cords (black arrow). H&E 40×. Inset: detail of the coelomyarian musculature and a uterus with ova from another section of another parasite of the same genus and species

During histopathological analysis of adult parasite sections, there are several key points to consider (Fig. 6). The first characteristic is the presence of a body cavity (pseudocoelom), which is absent in parenchymatous parasites such as trematodes or cestodes (Fig. 7a and b, respectively). Moreover, there are specific structures in cestodes, such as the presence of segmented bodies and calcareous corpuscles, which allow them to be differentiated from trematodes [47]. The presence of a digestive tract is also a histological characteristic that differentiates parasites, since acanthocephalans (Fig. 7c) and cestodes lack these structures [40].

Some features are typical of certain parasitic groups. For instance, the presence of articulated appendages and striated muscle in arthropods (Fig. 7d) [48], the observation of an anterior end with cephalic hooks in acanthocephalans [40] and the presence of a cuticle with sclerosed openings, head with hooks and acydophilic glands of pentastomids (Fig. 7e), which, among other structures, allows them to be differentiated from nematodes (Fig. 7f) [47]. Figure 6 shows a basic diagnostic algorithm with some of the main histological characteristics to consider when differentiating among several parasitic groups. After identifying these main morphological characteristics, the identification of other histological structures can be further explored, such as the presence of specializations of the cuticle and the hypodermis, and type of musculature, which can be holomyarian, polymyarian or meromyarian depending on the number of cells per nematode quadrant or coelomyarian or platymyarian according to the projection of muscle cells to the pseudocoelom. In addition, the type of cells of the digestive tract and characteristics of the reproductive tract are highly relevant for their taxonomical classification. It is important to highlight that, to achieve a more detailed determination at the genus and species levels, taxonomic dichotomous keys should be run [47].

### Interrelations between taxonomy and ecology

The relationship among taxonomy, ecology and biogeography is bidirectional. Incorrect species identification can lead to inaccurate ecological and biogeographical knowledge, while a lack of ecological information can hinder reliable species classification in the field. The absence of taxonomic resolution at the species level can impede our understanding of community-level phenomena and evolutionary processes [49].

The reliance on a few difficult-to-access experts in parasite taxonomy, coupled with the discovery of potentially new parasite species and international regulations on biological material exchange, complicates the process of species identification [50]. Cryptic species are common in many parasitic groups of veterinary and medical importance, often displaying genetic divergence and ecological niche differences with significant differences in their pathological effects [15]. Additionally, as field researchers cannot feasibly extract all specimens under study for molecular analysis, it is essential to recognize potential species differences based also on ecological traits, behavior and biogeographical distribution. An interesting example is provided with *Anisakis simplex, Anisakis pegreffi* and *Anisakis berlandi*, which were considered a single cryptic entity. Using allozyme and gene sequence analysis, this species complex was resolved into three separate taxa [51]. Since then, a vast description of *Anisakis* spp. genetic diversity in different hosts and geographical locations has been described [52].

For example, Ricklefs et al. [53] demonstrated the importance of ecological data in taxonomy by showing how host and vector ecology, biogeography and molecular information shape the distribution and diversity of hemosporidian parasites in birds. Similarly, Kincaid-Smith et al. [54] highlighted the role of ecological factors in the hybridization of European schistosome species, with significant implications for epidemiology and disease control. Those hybridized schistosomes, recognized by a combination of morphological, molecular and ecological evidence, resulted in hybrids with a capacity to infect a broader range of hosts, including humans and domestic animals, making their control more difficult.

Classification of parasites also considers biogeographical and ecological components. For instance, *Echinococcus* spp. identification is challenging, with many different genotypic groups among hosts and geographic locations. Studies in this group have been important to elucidate relationships between specimens from different regions of the world and the role of canids and other hosts in species differentiation as well as transmission and epidemiological risks of these species. The study of ecological divergence among cryptic species of *Echinococcus*, and combining ecological data with molecular analysis, has led to the recognition of distinct species with significant implications for taxonomy and epidemiology [55]. Moreover, the taxonomy of *Leishmania* spp., the parasites responsible for leishmaniasis, has been informed by ecological studies on vector distribution and environmental factors. Different *Leishmania* spp. are transmitted by specific sandfly species, which inhabit specific ecological regions. This in turn provides insights into the importance of some ecological factors influencing transmission of *Leishmania* spp., molecular mechanisms in the parasite-vector interactions and implications for disease control [56]. Another example includes macroscopically identical *S. lupi* worms found in domestic and wild canids and *Spirocerca vulpis* described in red foxes [57]. Having information about the host use and geographical distribution allows a first taxonomical differentiation between both species when microscopic methods are not readily available. However, additional tools should be used for their classification, since different host use led to the description of *Ascaris suum* as a different species of *Ascaris lumbricoides*, just because the former parasitized pigs rather than humans [58].

These examples show that ecological and biogeographical data are essential for accurate species delimitation, particularly in parasites. Integrating this information into taxonomic practices enhances our understanding of biodiversity, informs conservation efforts and improves the study of host-parasite interactions. Therefore, it is crucial to incorporate as much ecological and biogeographical information as possible when identifying or characterizing a new species from an integrative taxonomy perspective.

The use of geographical information systems (GIS), remote sensing (RS) and the application of ecological tools are useful for gaining knowledge in spatial parasitology. The information obtained in this new field, in turn, has eased the visualization of disease prevalence, case outbreaks and communication of the risk of infection [59]. However, GIS and RS go beyond disease cartography and have improved the monitoring of disease spread, the modeling of prevalence and the identification of disease clusters [60]. For instance, ArcGIS (www.arcgis) and QGIS (www.qgis) are just two examples of software routinely used for mapping the above situations. In addition, environmental, climatic, socio-economic and demographic data such as temperature, relative humidity, altitude, forest cover and degree of urbanization can be added to parasite dynamics data by using Bayesian spatiotemporal models [61]. It has been observed that spatial parasitology studies in human-related helminthiases have included cluster analysis, ecological niche modeling and endemicity, risk and prevalence mapping mainly in national-scale studies [59]. Nevertheless, these analyses greatly depend on the availability of complete and updated databases with environmental, demographic and parasite-related information. In this sense, the information accessible at the NCBI and the Global Biodiversity Information Facility (GBIF) may be used as complementary databases to study spatially georeferenced parasites [62]. Altogether, the analysis of the ecology of parasites is key to untangling their epidemiology, spatial distribution, pinpointing competent hosts and understanding their diversity and potential gene flow between isolates, as done for the apicomplexan *Hepatozoon canis* [63].

### DNA analysis

Obtaining DNA barcodes from mitochondrial or ribosomal loci has been easily accessible since the 1990s, which raised the question of whether the information derived from DNA sequences would supplant the knowledge obtained by traditional taxonomy methods, thereby prompting the adoption of integrative taxonomy [2, 3]. Molecular analyses have not supplanted traditional methods but have complemented them in the interpretation of cryptic diversity, presence of genotypes, delimitation of species, new species description and definition of species complexes [15, 64].

The value of samples destined for molecular studies starts from the moment these are preserved until sequence analysis. At all times, samples should be kept free from formalin, as this creates crosslinks of DNA sequences to proteins and deaminates and depurinates nucleic acids, thus resulting in DNA shearing and interference with subsequent DNA amplification [65]. Therefore, samples should always be maintained in ethanol at ≥ 70% to preserve parasite's eggs, larvae or adult tissues. At this point, samples should be primarily destined for molecular analysis since prolonged storage in ethanol results in dehydration and coiling of specimens [64], which will hinder morphological assessment. Adult specimens can be maintained in ethanol for years while keeping good DNA integrity. For instance, helminth barcodes have been successfully obtained from gastrointestinal tracts of shrews preserved in different ways in a natural history museum [66] or as demonstrated with the DNA amplification of specimens collected from red foxes kept at − 20 °C for over 20 years [67].

Fecal samples with eggs can be stored at − 80 °C with or without ethanol for months, years or even decades. Nevertheless, ethanol must be removed from tissue or fecal samples right before DNA extraction, since traces of this chemical may inhibit subsequent PCR reactions. Analyses on hookworm DNA amplification demonstrated that fecal samples without any preservatives may be stored at 4 °C for 60 days, at 32 °C for 10 days or at − 20 °C or − 80 °C for months without significant DNA degradation [68]. Importantly, coprological analyses aimed at the observation and collection of eggs and larvae should always be done before extracting DNA for single-locus amplification or metabarcoding analyses [69]. This warrants that any parasitic group found during DNA studies is accompanied by the microscopical confirmation of the respective stages, thereby discarding DNA contamination. Another point to consider is the possibility of spurious parasitism. This occurs when the detection of a certain parasite species, whether through morphological or molecular methods, is actually the result of predation of an animal with true parasitism and not related to infection of the host [70].

DNA can be extracted from helminths using commercial kits with column purification or “manual” protocols such as phenol-chloroform-isoamyl alcohol, ethanol DNA precipitation, freeze-thawing or heat treatment [71]. In addition, other methods like extraction in TE buffer and Chelex resin have also been tested without much success in plant-associated nematodes [72]. It is important to highlight that protocols designed for soil bacterial communities are also applicable to the analysis of helminths associated with fecal samples or scats due to the presence of buffers that prevent DNases and other PCR inhibitors from hindering subsequent analyses [16]. Therefore, special kits designed for stool or soil can be used to extract egg DNA following some modifications [41, 73]. These commercial kits are usually designed to extract bacterial DNA, which in most cases does not have a thick wall to protect these cells from dissecation. Contrarily, nematode eggs adjust to an hexalaminar model including a vitelline or *pellicular ovi*, a chitinous and a chondroitin-proteoglycan layer [74], thus highlighting the ultrastructural complexity of eggs and the need for additional steps for successful DNA extraction. Similarly, the trematode and cestode egg structure is also highly complex, with the presence of keratin, elastin or sclerotin layers in trematodes [75] and a proteinaceious shell and embryophore in cestodes [76]. Thus, adding glass beads or pearls to fecal samples with subsequent vortexing allows the mechanical lysis of the outer layer of helminth eggs. This step followed by chemical lysis with lysis buffers enables DNA derived from eggs to be released [77]. The same kits for fecal or soil samples with their modifications should be followed if larval DNA is analyzed [78].

DNA extraction from adult stages of nematodes, trematodes and cestodes can be done using the “manual” protocols mentioned above, but commercial kits designed for tissues have the best performance in terms of DNA final purity [41, 79]. Extraction protocols usually require 50 mg of tissue; therefore, a 1-cm section of a 2–4-mm-wide adult nematode, half of a cestode proglottid or half trematode body are used. Of course, divergence in sizes occurs between species and therefore whole small individuals [79] or even a 1-mm transversal section of a large nematode can be used. If larger amounts of tissues are used, columns will get clogged, and further purification and amplification steps will fail.

Different parts of a helminth’s body will give different results depending on the analysis. For instance, if amplification of a few mitochondrial or ribosomal loci is the aim, then any body portion will have the same value. However, if whole-genome studies are conducted, then body sections from a female uterus should be avoided since it will generate genome and mitochondrial chimeras with the egg's different genetic material and the female's somatic cells [80]. If only a few specimens are available for an integrative taxonomy analysis, the middle portion of a nematode's body should be used to keep anterior and posterior ends available for morphological assessment. Similarly, half of a trematode's body or a proglottid can be used for DNA extraction and the other half for staining and taxonomic analysis.

**Fig. 8:**
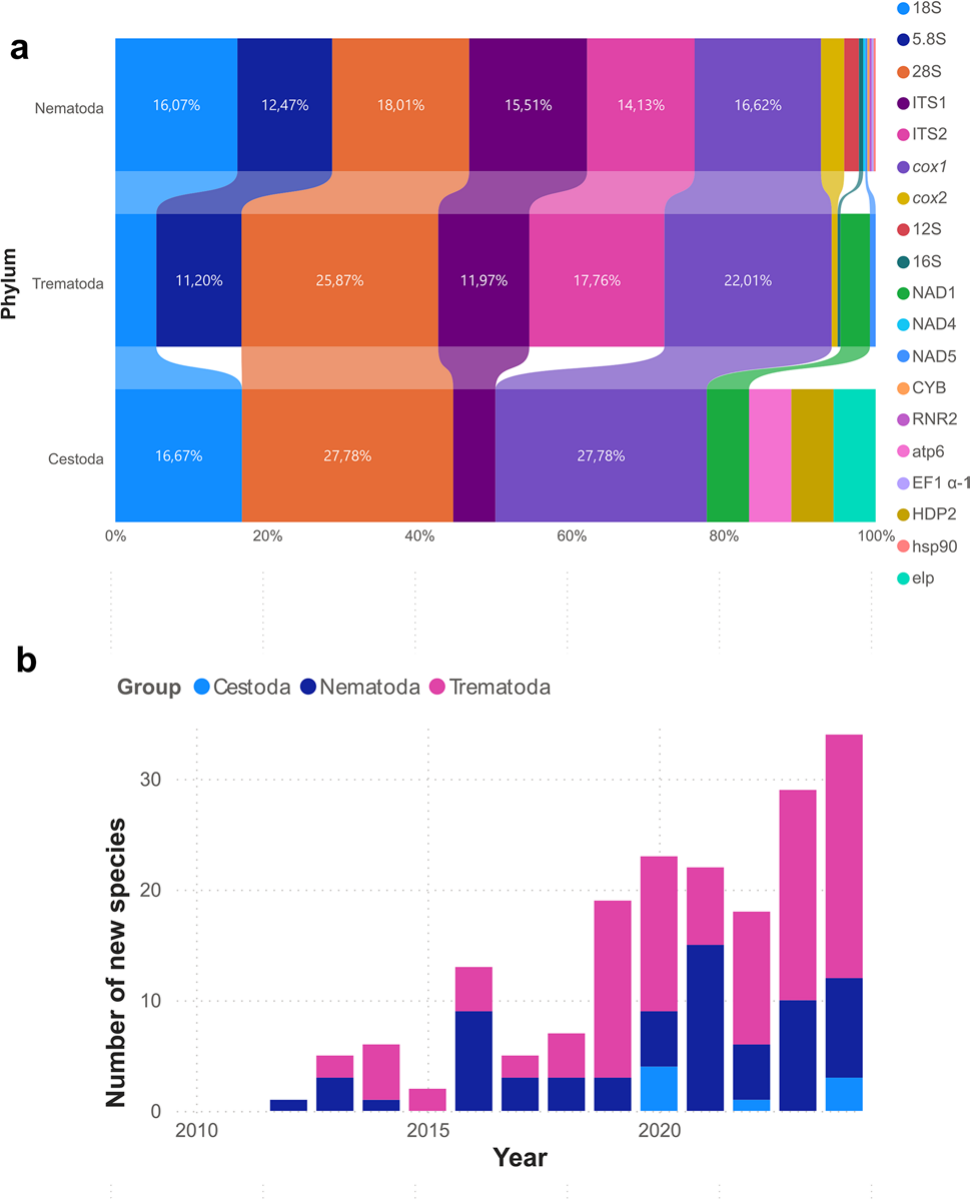
Use of different DNA markers **a** and description of new species **b** using integrative taxonomy approaches

Once DNA is extracted, PCR will be conducted targeting different loci depending on the parasitic group and family (Table 1). For instance, mitochondrial and ribosomal markers are among the most employed targets due to their high interspecies variation, which is essential to delimitate species (Fig. 8b) [81]. Ideally, several loci should be employed to reach the identification of a specimen by the combination of ribosomal, mitochondrial or nuclear markers. Among these, ribosomal rDNA like the 18S rDNA or ribosomal small subunit (SSU) is useful for providing the higher taxonomic status of an unknown specimen. Furthermore, internal transcribed spacers 1 (ITS1) and 2 (ITS2) accumulate a higher number of mutations [82] and therefore, provide high inter- and intraspecies variations useful for identity confirmation and population genetic studies [83]. Interestingly, the 28S rDNA has larger interspecies variation than the 18S rDNA but lower than ITS regions at the order, family, genus and species levels of nematodes, trematodes and cestodes [84]; therefore, these regions have been widely used.

**Table 1:** Primer examples used for the amplification of conserved ribosomal or mitochondrial DNA regions of helminths

Targeted group	Locus	Fragment size	Primer sequence	References
All metazoans	*cox*1	~ 710 bp	LCO1479 (5'-GGTCAACAAATCATAAAGATATTGG-3') HCO2190 (5'-TAAACTTCAGGGTGACCAAAAAATCA-3')	[108]
Nematoda	ITS1, 5.8S, ITS2, 28S	~ 1500 bp	652 (5'-GCAGCCGCGGTAATTCCAGCTC-3') D3b-R (5'-TAGTAGCTGGTTCCTTCGCA-3')	[109]
Nematoda	28S	~ 400 bp	D2A (5'-ACAAGTACCGTGAGGGAAAGTTG-3') D3b (5'-TCGGAAGGAACCAGCTACTA-3')	[109]
Nematoda and Cestoda	*cox*1	~ 395 bp	JB3 (5'-TTTTTTGGGCATCCTGAGGTTTAT-3') JB45 (5'-TAAAGAAAGAACATAATGAAAATG-3')	[110]
Nematoda	ITS1	400–800 bp	rDNA2 (5'-TTGATTACGTCCCTGCCCTTT-3') rDNA158S (5'-ACGAGCCGAGTGATCCACCG-3')	[111]
Nematoda	18S	~ 1000 bp	Nem18S-F (5'-CGCGAATRGCTCATTACAACAGC-3') Nem18S-R (5'-GGGCGGTATCTGATCGCC-3')	[112]
Nematoda	18S	650 bp	Nem1 (5'-GCAAGTCTGGTGCCAGCAGC-3') Nem2 (5'–3')	[113]
Nematoda	ITS1	400–800 bp	BL18 (5'-CCCGTCGMTACTACCGATT-3') 5818 (5'-ACGARCCGAGTGATCCAC-3')	[114]
Nematoda (Clade V)	ITS2	~ 600 bp	NC1 (5'-ACGTCTGGTTCAGGGTTGTT-3') NC2 (5'-TTAGTTTCTTTTCCTCCGCT-3')	[115]
Nematoda	12S	~ 460 bp	12S.C345.F (5'-GTWCCAGAATAATCGGMTA-3') 12S.C345-R (5'-ATTGAYGGATGRTTTGTRC-3')	[81]
Nematoda	16S	~ 240 bp	16S.C345.F (5'-AAGATAAGTCTTYGGAARYT-3') 16S.C345.R (5'-GAAYTAAACTAATATCAMG-3')	[81]
Trematoda	ITS2	~ 400 bp	3S (5'-GGTACCGGTGGATCACTCGGCTCGT-3') A28 (5'-CCTGGTTAGTTTCTTTTCCTCCGC-3')	[110]
Trematoda	ITS2 and a portion of the 5.8S	~ 450 bp	3S (5'-GGTACCGGTGGATCACTCGGCTCGT-3') BD2 (5'-ATCTAGACCGGACTAGGCTGTG-3')	[116]
Trematoda	18S	~ 600 bp	Trem-18SF (5'-ATGGCTCATTAAATCAGCTAT-3') Trem-18S-R (5'-TGCTTTGAGCACTCAAATTTG-3')	[117]
Trematoda	28S	~ 600 bp	LSU5 (5'- TAGGTCGACCCGCTGAAYTTAAGC-3') 1500R (5'- GCTATCCTGAGGGAAACTTCG -3')	[118]
Trematoda	28S	~ 1200 bp	Dig12 (5′-AAGCATATCACTAAGCGG-3′) 1500R (5′-GCTATCCTGAGGGAAACTTCG-3′)	[119]
Cestoda	18S	~ 260 bp	Cest3 (5'-YGAYTCTTTTTAGGGGAAGGTGTG-3') Cest5 (5'-GCGGTGTGTACMTGAGCTAAAC-3')	[120]
Cestoda	18S	~ 1800 bp	WormA (5'- GCGAATGGCTCATTAAATCAG-3') WormB (5'-CTTGTTACGACTTTTACTTCC-3')	[121]
Cestoda	*nad*1	529 bp	JB11 (5'-AGATTCGTAAGGGGCCTAATA-3') JB12 (5'-ACCACTAACTAATTCACTTTC-3')	[116]
Cestoda	*cox*1	570–585 bp	Dice1F(5'-ATTAACCCTCACTAAATTWCNTTRGATCATAAG-3') Dice11R (5'-TAATACGACTCACTATAGCWGWACHAAATTTHCGATC -3')	[122, 123]
Cestoda	28S	~ 1660 bo	ZX-1 (5'-ACCCGCTGAATTTAAGCATAT -3') 1500R (5'-GCTATCCTGAGGGAAACTTCG-3')	[121]
Cestoda	*nad*1 + trnN	~ 850 bp	Cyclo-nad1F (5'-GGNTATTSTCARTNTCGTAAGGG-3') Cyclo-trnNR (5'-TTCYTGAAGTTAACAGCATCA-3')	[121]
Cestoda	16S	~ 400 bp	Cyclo16S-F (5'-TGCCTTTTGCATCATGCT-3') Cyclo16S-R (5'-AATAGATAAGAACCGACCTGG-3')	[121]

Mitochondrial genes are encoded as multiple copies, are maternally inherited and have low recombination rates and higher mutation rates than protein coding rDNA genes [85]. For all these characteristics, these loci, especially the cytochrome oxidase subunit 1 (*cox*1), have been used for barcoding purposes. In addition, *cox*1 has the largest repository of sequences in databases as demonstrated with three nematode families of clinical and veterinary importance [83]. Similarly, 12S and 16S mitochondrial rDNA genes have comparable nucleotide differences compared to *cox*1 but have slower evolution rates than protein-coding mitochondrial genes [83], making them ideal for metabarcoding analyses [81]. In the phylum Nematoda, the 28S rDNA, *cox*1 and 18S rDNA genes are among the most used, followed by ITS1 and ITS2 in integrative taxonomy studies (Fig. 8b). Moreover, 28S rDNA, *cox*1 and ITS2 regions are the most employed loci in trematode analysis, whereas in cestodes *cox*1, 28S and 18S rDNA genes are the most used.

Obtained amplicons should be compared to publicly available sequences in databases. Generally, 97% species identity is desired for mitochondrial and ITS regions as seen in numerous studies [86–88]. However, there is no yardstick to define the differences between species, since this will depend on the marker and the parasitic group [89]. For instance, genetic distance between species in the 18S rDNA gene is < 0.007% in nematodes, 0.014% for trematodes and 0.030% in cestodes, whereas the nucleotide distance in the *cox*1 gene is 0.12%, 0.17% and 0.12%, respectively [84]. Therefore, 99% sequence identity in an 18S rDNA sequence does not lead to species identification, whereas this same value for the *cox*1 sequences does, as demonstrated previously [90].

Inference of the evolutionary history of worms, their relationships and possible geographical or host origin of the collected specimens may be inferred with phylogenetic, genetic distance and haplotype network analyses. First, sequences need to be aligned with similar ones available in databases using software like MEGA [91] or MAFFT [92].Accordingly, phylogenetic trees can be estimated with neighbor-joining, maximum likelihood and Bayesian inference trees [83, 93] using software like MEGA, MrBayes [94], the BEAST package [95], IQ-Tree [96] or Geneious (www.geneious.com) and visualized using FigTree, Treeviewer [97] and iTOL [98], among others. Several points should be considered when building trees, including the chosen outgroup, the best nucleotide substitution model, the number of bootstraps and posterior probabilities in ML and BI trees, respectively, to test the robustness of the trees, and the analysis of sequences preferentially > 300 bp. Finally, haplotype networks generally draw the connection between two or more sequences by separating each sequence with mutational steps and hypothetical haplotypes. For this purpose, PopArt [99] and Network use different algorithms such as minimum spanning, median joining, ancestral maximum parsimony, interger neighbor joining, tight span walker or Templeton Crandall Sing.

### Specimen identification and dichotomous keys

Given the complexity of species definition, attempting to establish a precise set of characters that define a species can be a lifelong task. Pure taxonomic efforts alone may not suffice to accomplish this for every parasitic subgroup. A more practical approach could be to view species as separately evolving lineages, defined by one or more sources of evidence. As we gather more data—morphological, ecological, molecular, biogeographical and behavioral—a more reliable species delimitation can be achieved [1]. This shifts the focus from defining species by a fixed set of characters to considering the evidence that supports an organism's classification as a distinct lineage [100, 101].

For parasites, many of which have complex life cycles with multiple morphologically distinct stages, an integrative approach to taxonomy should include as much ecological and epidemiological information as possible for accurate species identification and delimitation. The speciation continuum theory unifies these ideas by defining species based on a range of traits—morphological, genetic and ecological—in response to selective pressures and other factors [102, 103]. When these traits demonstrate that specimens belong to a separately evolving lineage, a species can be delimited. After morphological and histopathological evidence has been collected, key morphological traits should be considered for their identification (Fig. 6). These data together with ecological features and percentages of DNA identification often lead to the diagnosis of the specimen, the description of a new taxonomic entity or the finding of cryptic diversity (Fig. 8b).

Dichotomous keys have been used for the classification of specimens to the family level, like the one from Anderson, Chabaud and Willmott for the phylum Nematoda [104], the guide proposed by Gibson, Bray and Curtis for the class Trematoda [105] and Khalil, Jones and Bray for the class Cestoda [106]. However, more specific keys are available for each family, which are regularly updated according to the description of new species and taxonomic reclassifications. It is crucial that researchers can recognize each morphological structure with either light microscopy or SEM so dichotomous keys are used properly. This is the most difficult task to accomplish and usually requires skills collected from an entire career as a taxonomist. Therefore, morphological identification may cease to exist if interest in taxonomy diminishes in future generations [12]. If doubts arise, we recommend contacting taxonomists or collection curators from other countries so the specimens are correctly identified. Improper characterization may lead to the overrepresentation of helminth diversity and the creation of inaccurate epidemiological, pathological and ecological patterns.

The number of new species using integrative taxonomy approaches has increased in the last 15 years, especially in the Class Trematoda, with 230 new entities, followed by 67 new nematode species and eight new cestode species. These new species are usually the result of an exhaustive investigation of morphological, ecological, histopathological and molecular differences compared to reference specimens that finalize in the creation of a new taxon [107]. Nevertheless, when morphological and molecular differences are not conclusive for species classification, further analyses should be run. This could be because of improper preservation of specimens, leading to inadequate staining and contraction of structures or sub-optimal PCR conditions that cause non-resolved DNA sequences. Thus, sequencing different loci or staining additional specimens may decipher the taxonomic status of the collected specimen.

Cryptic diversity occurs when morphological and molecular evidence is inconclusive [15]. Cryptic species sensu stricto have large molecular divergence without evident morphological differences as confirmed by light microscopy or SEM. This occurs because of a speciation continuum that does not finish in two separated and evolving lineages [100] but rather analyzes specimens in the “gray zone” of speciation [15]. On the other hand, cryptic species sensu lato are suggested when only molecular data are available, but specimens cannot be morphometrically analyzed. In either case, proper care should be taken to run dichotomous keys so structures are properly identified for the specimen's characterization.

## Conclusions

Integrative taxonomy in helminthology has been increasingly used in the characterization of specimens for the diagnosis of an infection or the description of new taxonomic entities. This has led to the comprehensive analysis of worms considering not only morphological characters or DNA sequences but also the lesions caused in their hosts, their ecology and epidemiology. Nevertheless, the analysis of each aspect requires the integration of evidence to reach a proper diagnosis and necessitates the integration of different and sometimes divergent disciplines, which is key to successfully achieve scientifically sound results. Importantly, one discipline does not replace another one, as previously thought with molecular biology replacing systematics, but creates a synergy that has improved our understanding of helminth evolution and taxonomy.

## Supplementary Information

Additional file 1.

## Data Availability

Data used for the analysis of articles  with integrative taxonomy terms is available in Supplementary File 1.

## Abbreviations

*cox*1: Cytochrome oxidase subunit 1
CPD: Critical point drying
ITS: Internal transcribed spacer
CPD: Critical-point dryer
rDNA: Ribosomal DNA
SEM: Scanning electron microscopy
